# Difference in Long-Term Trends in COPD Mortality between China and the U.S., 1992–2017: An Age–Period–Cohort Analysis

**DOI:** 10.3390/ijerph16091529

**Published:** 2019-04-30

**Authors:** Haoyu Wen, Cong Xie, Lu Wang, Fang Wang, Yafeng Wang, Xiaoxue Liu, Chuanhua Yu

**Affiliations:** 1 Department of Preventive Medicine, School of Health Sciences, Wuhan University, #185 Donghu Road, Wuhan 430071, China; haoyuwen@whu.edu.cn (H.W.); ffdw03@whu.edu.cn (L.W.); wangfang0923@whu.edu.cn (F.W.); wonyhfon@whu.edu.cn (Y.W.); liuxx019@163.com (X.L.); 2 Hubei Center for Disease Control and Prevention, Wuhan, Hubei 430079, China; xxiiee0616@163.com; 3 Global Health Institute, Wuhan University, #8 Donghu Road, Wuchang District, Wuhan 430072, China

**Keywords:** COPD mortality, long-term trends, age–period–cohort model

## Abstract

Complications due to chronic obstructive pulmonary disease (COPD) is a leading cause of death in China and the United States (U.S.). This study aimed to investigate the long-term trends in COPD mortality in China and the U.S. using data from the Global Burden of Disease Study 2017 (GBD 2017) and explore the age, period, and cohort effects independently by sex under the age–period–cohort (APC) framework. Taking the age group 40–44 years old, the period 1992–1996, and the birth cohort 1913–1917 as reference groups, we found that the age relative risks (RRs) of COPD mortality increased exponentially in both China and the U.S., the period RRs increased in the U.S. but decreased in China; and the cohort RRs showed an overall downward trend in both China and the U.S. with the year of birth. From 1992 to 2017, the increased RRs of COPD mortality in the U.S. was mainly attributable to the increased prevalence of smoking before 1965, while the decreased RRs of COPD mortality in China was mainly attributable to reduced air pollution as well as improvements in medical technology and more accessible health services. Reducing tobacco consumption may be the most effective and feasible way to prevent COPD in China. However, we also need to pay more attention to COPD in nonsmokers in the future.

## 1. Introduction

Chronic obstructive pulmonary disease (COPD), a lung disease characterized by persistent airway obstruction [1], is a serious public health problem worldwide [2]. Complications due to COPD was the third leading cause of death in China since 1990 [3]. Chronic lower respiratory diseases was the third leading cause of death in the United States (U.S.) since 2008, and COPD is the primary contributor to mortality caused by chronic lower respiratory diseases [4]. Cancer and heart disease were the leading causes of death in the U.S. and China [5,6,7,8]. In 2017 alone, COPD caused 965.9 thousand and 169.3 thousand deaths in China and the U.S, respectively, accounting for one-third of the COPD deaths worldwide [9]. Although COPD mortality has shown a decreasing trend in China over the past 25 years, COPD mortality in China in 2017 was still higher than that in the U.S. [9]. Given the huge health burden caused by the disease, we need to urgently investigate the root causes of the trends in COPD mortality.

Previous research on COPD in China and the U.S. has mainly focused on its prevalence [10,11] and risk factors [12,13,14], while a few researchers have also looked at long-term trends in COPD mortality [15,16]. However, these studies were not age-adjusted, and none of them analyzed the reasons behind the long-term trends in COPD mortality in China and the U.S.

To address these limitations, this study aimed to investigate the long-term trends in COPD mortality in China using data from the Global Burden of Disease Study 2017 (GBD 2017) and explore the age, period, and cohort effects independently by sex under the age–period–cohort (APC) framework. By making a comparison with the U.S., we could find possible shortcomings in the prevention of COPD in China and learn from the advanced experience in reducing COPD mortality in the U.S.

## 2. Materials and Methods

### 2.1. Data Sources

This study applied the latest data from GBD 2017, which provides a comprehensive assessment of age- and sex-specific mortality for 282 causes of death in 195 countries and territories from 1990 to 2017 [17]. The study used five data sources to gather information about causes of death, and the data for COPD mortality were mainly extracted from two sources: the disease surveillance points system and the cause of death reporting system [18]. In the GBD program, data was reported based on the 9th and 10th revision of the International Classification of Disease (ICD). Age-standardized mortality from COPD in China and the U.S. were based on the GBD 2017 global age-standard population. GBD’s Cause Of Death Ensemble modeling (CODEm) tool was used to estimate COPD mortality more precisely, and the smoking prevalence, cigarettes per capita, proportion of population exposed to household air pollution, mean exposure to ambient particulate matter from outdoor air pollution, scale of the combined exposure to risks for COPD, and the Socio-Demographic Index (SDI) were included in the model as covariates [19].

### 2.2. Statistical Analysis

The APC model is a common sociological, demographic, and epidemiological model and is used to assess the age, period, and cohort effects independently on disease incidence and mortality rate. The three different time-related variations on disease incidence and mortality rate are distinguished in the APC model [20,21]. Age effects relate to variations associated with different age groups and represent age-related developmental changes and accumulation of exposures. Period effects are variations over time periods that affect all age groups simultaneously and represent changes in medical and diagnostic technology, disease classification, culture, and economy that are unique to a particular time period. Cohort effects relate to changes across groups of individuals who were born around the same time and represent early-life exposure to socioeconomic, behavioral, and environmental factors.

The application of the APC model has been plagued by multicollinearity problems since it was first proposed by Mason in 1973 [22]. The APC model can be considered essentially as a multiple regression model with the following expression:

Y = log(M) = μ + αX_1_ + βX_2_ + γX_3_ + ε (1)

where X_1_, X_2_, and X_3_ denote the age, period, and cohort, respectively, and M denotes the corresponding COPD mortality rate. α, β, and γ denote the coefficients of age, period, and cohort effects, respectively, as estimated by the APC model, while μ denotes the intercept and ε denotes the random error.

According to the definition of X_1_, X_2_, and X_3_, it is obvious that X_1_ = X_2_ − X_3_. This means that there is multicollinearity on variables X_1_, X_2_, and X_3_, and so we cannot get a uniquely determined solution in the APC model. In 2004, Yang first proposed the intrinsic estimator (IE) method to solve the multicollinearity problem of the APC model [23,24,25]. The IE method expresses the APC model equations in the following matrix form:

Y = Xb + ε(2)

where Y denotes the logarithm of COPD mortality by age, period, and birth cohort; X denotes the design matrix; and b denotes a vector composed of the age, period, and cohort effect coefficients.

The dimension of the design matrix is the full rank minus one due to the multicollinearity problem of age, period, and birth cohort. Therefore, according to the knowledge of linear algebra, the solution set P of Equation (2) can be expressed as a direct sum of N and Θ:

P = N ⊕ Θ(3)

where the one-dimensional solution set N can be written as {tB_0_} and has X∙tB_0_ = 0. It is obvious that B_0_ is completely determined by the matrix X. For an arbitrary solution, b ^ of the APC model can be written as follows:

b ^ = B + tB_0_ (4)

where B is a uniquely determined solution of the APC model, that is, an IE solution of the APC model.

To estimate the age, period, and cohort effects of COPD mortality using the APC model, age-specific COPD mortality rate was grouped into consecutive five-year periods from 1992 to 2017 and successive five-year age groups from 40–44 years to 75–79 years. The occurrence of death from COPD in the population aged under 40 years is rare and therefore not included in this study. The population aged over 80 years is recorded as one group in the GBD database. As this does not satisfy the data format of the APC model [21,23], they were also not used in this study. The data on COPD mortality from 1990 to 1991 was not included because it was not enough for a five-year period.

The coefficients of age, period, and cohort effects estimated by the APC model cannot be explained directly; therefore, we calculated the relative risk (RR) to help explain the age, period, and cohort effects independently on COPD mortality. Taking the age group 40–44 years old, the period 1992–1996, and the birth cohort 1913–1917 as reference groups, we calculated the difference in parameter estimations between other groups and the reference group, and the exponent of the difference was taken as the corresponding RR value. In this study, APC analyses were implemented using the Stata 12.0 software (StataCorp, College Station, TX, USA). In addition, we took a Wald test on the results of the APC model and considered *p* < 0.05 as statistically significant. The Akaike information criterion (AIC), the Bayesian information criterion (BIC), and deviance were used to check the degree of model fitting.

## 3. Results

### 3.1. The Overall Trends in COPD Mortality in China and the U.S.

**Figure 1:**
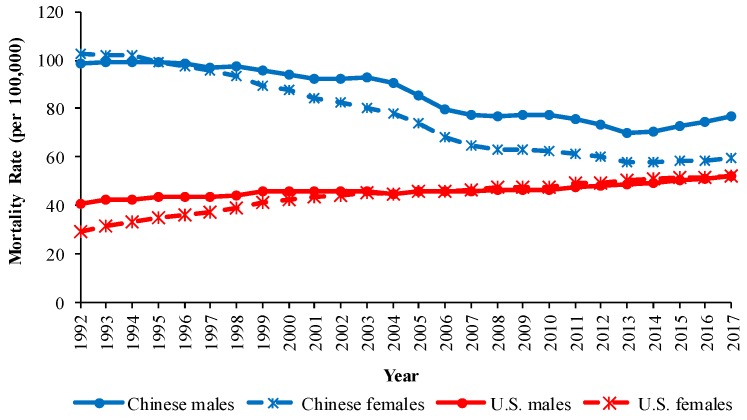
Trends in crude mortality rates (CMRs) from chronic obstructive pulmonary disease (COPD) by sex in China and the U.S., 1992 to 2017.

**Figure 2:**
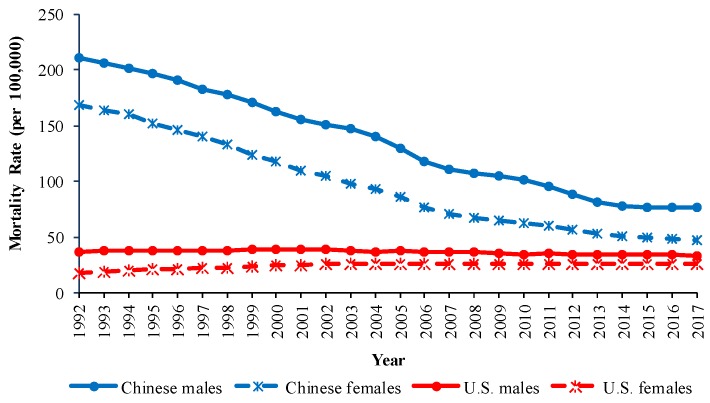
Trends in age-standardized mortality rates (ASMRs) from COPD by sex in China and the U.S., 1992 to 2017.

Trends in the crude mortality rates (CMRs) and the age-standardized mortality rates (ASMRs) for COPD by sex in China and the U.S. from 1992 to 2017 are shown in Figure 1 and Figure 2. From 1992 to 2017, the CMRs of COPD showed a downward trend in China but showed an upward trend in the U.S. The ASMRs of COPD in China showed a significant downward trend from 1992 to 2017, while the ASMRs changed little from 1992 to 2017 in the U.S. In 2017, Chinese males had the highest ASMR, followed by Chinese females, U.S. males, and U.S. females.

### 3.2. The Variation in Age, Period, and Cohort on COPD Mortality

**Figure 3:**
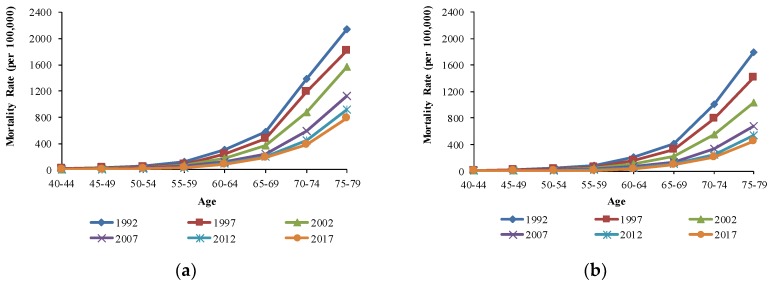

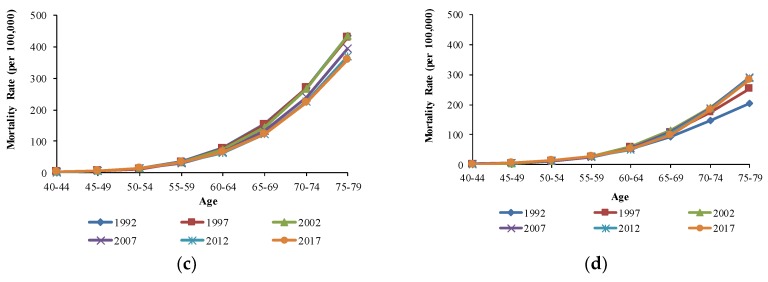
Age-specific COPD mortality rate for (**a**) Chinese males, (**b**) Chinese females, (**c**) U.S. males, and (**d**) U.S. females from 1992 to 2017.

Trends in the age-specific mortality rates from COPD by sex in China and the U.S. from 1992 to 2017 are shown in Figure 3. In both countries, age-specific COPD mortality showed a similar pattern of exponential increase with age. For the same age group, the age-specific COPD mortality showed a downward trend with period in China, and this trend was more significant in the older age groups. Unlike China, the age-specific COPD mortality changed little with period in the U.S.

**Figure 4:**
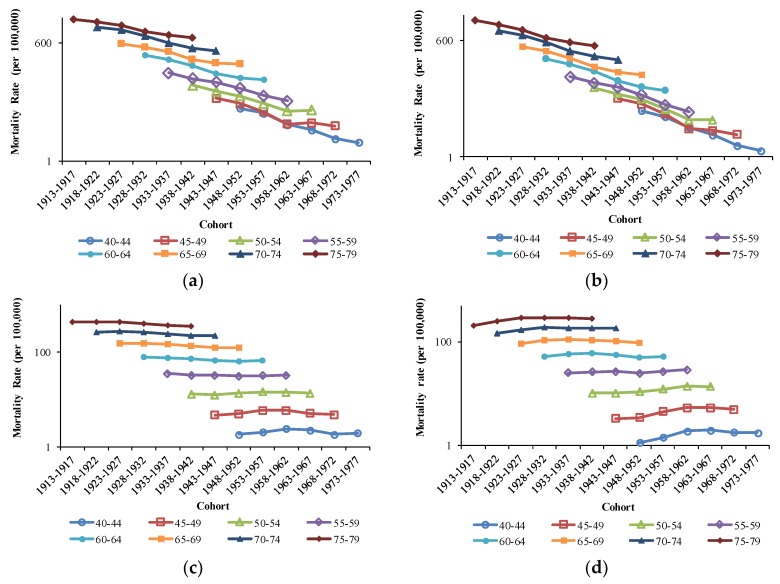
Cohort-based variation in age-specific COPD mortality in (**a**) Chinese males, (**b**) Chinese females, (**c**) U.S. males, and (**d**) U.S. females.

The cohort-based variations in age-specific COPD mortality are shown in Figure 4. The COPD mortality is plotted in log scale in order to more clearly show the impact of cohort in lower age groups. For the same age group, age-specific COPD mortality rates in China showed a downward trend with the birth cohort, and this trend was more significant in the older generation. For U.S. males, age-specific COPD mortality rates decreased slightly with the birth cohort overall, while the age-specific COPD mortality increased first and then decreased with the birth cohort for U.S. females. However, the cohort in this study spanned 65 years, and the age and period effects on COPD mortality were confounded, meaning the cohort effects could not be shown independently. As a result, we needed to use the APC model to assess the age, period, and cohort effects independently on COPD mortality.

### 3.3. The Age, Period, and Cohort Effects on COPD Mortality

**Figure 5:**
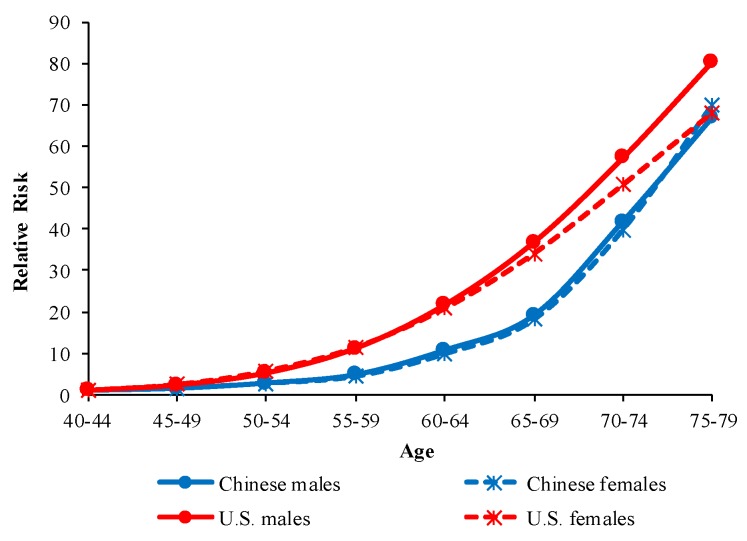
Age effects on COPD mortality by sex in China and the U.S.

The age RRs of COPD mortality in China and the U.S. are displayed in Figure 5. After further curve estimation, we found that the RRs of age effects in China and the U.S. followed exponential distributions (*R^2^* = 0.9953 for Chinese males, *R^2^* = 0.9935 for Chinese females, *R^2^* = 0.9795 for U.S. males, *R^2^* = 0.9708 for U.S. females).

**Figure 6:**
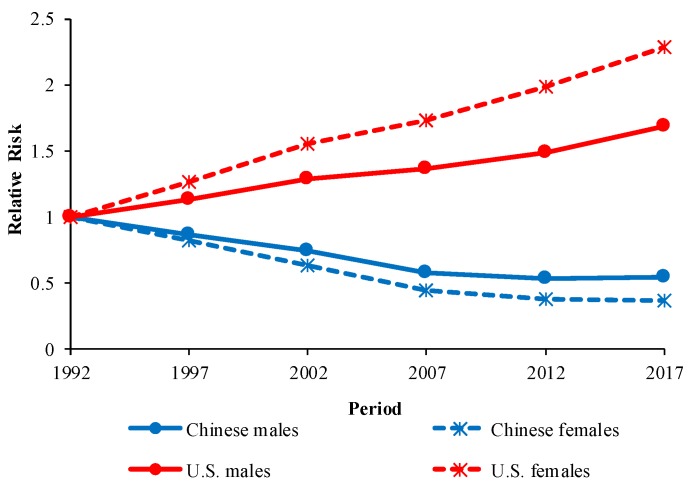
Period effects on COPD mortality by sex in China and the U.S.

The period RRs of COPD mortality in China and the U.S. are displayed in Figure 6. In China, the period RRs showed a downward trend for both sexes, whereas they showed an upward trend for both sexes in the U.S.

**Figure 7:**
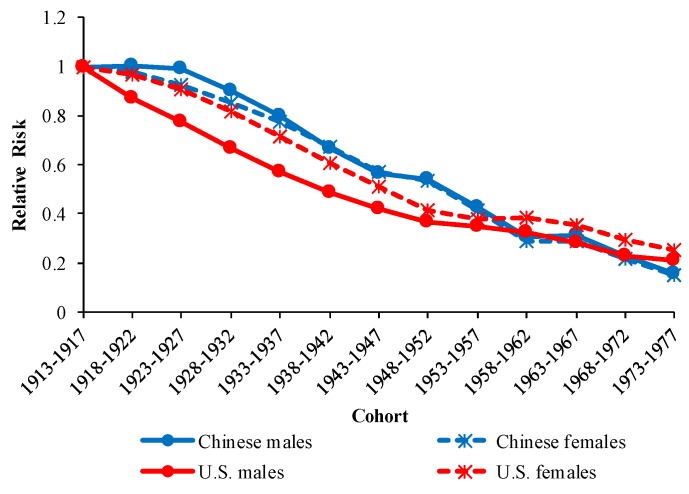
Cohort effects on COPD mortality by sex in China and the U.S.

The cohort RRs of COPD mortality in China and the U.S. are displayed in Figure 7. The cohort RRs in China and the U.S. showed a downward trend with the birth cohort overall. The downward patterns of cohort RRs differed between China and the U.S. but were similar between males and females for the same country. The downward trend in cohort RRs in China slowed down in the cohorts 1948–1952 and 1963–1967, while it slowed down in the cohorts 1953–1957 and 1958–1962 in the U.S. The estimated coefficients; RRs of age, period, and cohort effects; the *p* values; AIC; BIC; and deviance are all shown in the Appendix A.

## 4. Discussion

COPD is the third leading cause of death in China and the U.S., and understanding its mortality trends and features are therefore of great significance. This study investigated the long-term trends in COPD mortality in China and the U.S. and explored the age, period, and cohort effects on COPD mortality independently by sex using the IE method of the APC model. Findings from this study could provide theoretical support for the prevention of COPD in the future.

Age effects reflect the impact of physiological changes caused by aging and accumulated exposure to risk factors on COPD mortality. After adjusting for period and cohort effects, the RRs of COPD mortality increased exponentially with age in both China and the U.S. Physiological changes caused by aging result in a decline in immunity and lung function. Lung function plays an important role in the development of COPD [26,27]. Previous studies have suggested that lung function peaks at the young life stages and begins to decline at the age of 25 [28]. The decline in immunity makes elder people more vulnerable to air pollution and increases the risk of death from COPD [29]. The accumulation of exposure to risk factors in the elderly population has been proven to be associated with the development of COPD [30,31]. In addition, the limitation in activity in older people increases the difficulty of providing COPD treatment, therefore increasing the risk of COPD mortality. According to estimations by the United Nations, demographic aging in China and the U.S. will be more serious in the future. In 2017, the proportion of people over 60 years in China and the U.S. was 16.2% and 21.5%, respectively. This is expected to increase to 25.1% and 25.9% by 2030 and to 35.1% and 27.8% by 2050 [32]. The burden of COPD mortality can be expected to become more serious in the future, and we need to therefore pay more attention to the prevention of COPD.

Period effects represent changes in medical technology, economics, and culture that are unique to a particular period. From 1992 to 2017, the period RRs of COPD mortality increased in the U.S. but decreased in China. Before analyzing period effects on COPD mortality, we should have confirmed the impact of the revision of ICD on this research. However, previous studies have proven that the change from ICD-9 to ICD-10 had no substantial impact on the study of long-term trends in COPD mortality [33].

The increased period RRs in the U.S. over the past 25 years were mainly attributable to the increased smoking prevalence in the 1960s. Tobacco smoking has long served as the most important risk factor for COPD in the U.S. [34]. In the early 20th century, the smoking prevalence in the U.S. increased rapidly due to the promotion of cigarettes by mass media and improvements in large-scale tobacco production as well as the liberalization of female roles [35]. Smoking prevalence peaked in the 1940s and 1950s for males in the U.S., while it peaked in the 1960s for females [36]. Several studies have suggested that tobacco smoking holds long lag effects on COPD death [16,37]. Increased smoking prevalence before 1965 played a big contribution to the increased period RRs of COPD mortality in this study, which is consistent with several studies looking at the trends in COPD mortality in the U.S. [16,38] In 1964, a landmark report by the Surgeon General’s Advisory Committee on Smoking and Health was published [39], and the smoking prevalence in the U.S. declined continuously in the later decades. In 1965, the smoking prevalence in U.S. adults was 42.4%, but this had decreased to 14.0% by 2017. The proportion of ever-smokers who have quit has also increased [40]. In view of the lagged effects of tobacco smoking on COPD death, we can expect that the risk of COPD mortality will decline within the next decades. Tobacco smoking is also an important risk factor for COPD in China [15]. Due to the limitations in accessing data, we were unable to track the smoking prevalence in China before 1984. However, data after this date is available. National Health Service Surveys show that the smoking prevalence in China had a slight upward trend from 33.88% in 1984 to 35.3% in 1996 [41,42]. It declined to 26.0% in 2003 but changed only slightly to 25.2% in 2013 [43,44]. The database of the World Bank shows the smoking prevalence in China was 25.6% in 2016 [45]. Considering the lagged effects of tobacco smoking on COPD mortality, the health burden of COPD caused by tobacco smoking is likely to increase first in the near future, then decline for a period, and finally maintain a stable level. However, the continued high smoking prevalence in China deserves more attention. Compared with the U.S., the smoking prevalence in China can be greatly reduced. Considering the fact that China is the country with the largest number of active smokers in the world [46] and taking into account the huge impact of tobacco smoking on COPD mortality, reducing tobacco consumption may be the most effective and feasible measure to prevent COPD in China. It must be noted that a substantial number of COPD patients in both China and the U.S. have never smoked [47,48]. However, there have been limited studies on the trend in COPD patients who have never smoked. With improving smoking behavior in both China and the U.S., further research on COPD in nonsmokers is urgently needed.

Change in the definition of COPD is also a source of the upward trend in period RRs in the U.S. Historically, COPD was defined by the presence of certain symptoms, such as cough and sputum production, based on self-reported data. This led to potential underdiagnosis in elderly people and patients with mild COPD [34,49,50]. The Global Initiative for Chronic Obstructive Lung Disease (GOLD) in 2002 allowed doctors to diagnose COPD based on pulmonary function tests so that COPD patients could be diagnosed more accurately [51]. There was a large number of undiagnosed patients with COPD in the U.S. based on the original COPD definition [49,52], and the estimates of COPD prevalence based on pulmonary function tests may be as much as double the estimates based on self-reported data in the U.S. [50,53]. Improvements in the COPD diagnostic method, such as spirometric screening, lung diffusion capacity test, chest radiograph, and arterial blood gas test, have also helped to diagnose COPD patients more accurately [10,50,54]. In China, the change in the definition of COPD also affected a lot of undiagnosed patients with COPD, with the estimates of COPD prevalence based on pulmonary function tests as much as triple the estimates based on self-reported data [55]. However, under the influence of improved medical technology and more accessible health services as well as reduced air pollution, period RRs in China declined from 1992 to 2017.

Improvements in medical technology and more accessible health services contributed a lot to decreased period RRs of COPD mortality in China in the past 25 years, which is consistent with the finding of another study conducted by Yin et al. [15]. Improved treatment, for example, roflumilast and salmeterol/fluticasone propionate, also facilitated reduced mortality in Chinese COPD patients [56,57]. Reforms in the Chinese healthcare system has enabled more people to benefit from improved medical technology. In China, the Basic Social Medical Insurance for Urban Employees started in 1998, the New Rural Cooperative Medical Insurance Scheme was launched in 2003, and the Basic Social Medical Insurance for Urban Residents started in 2007 [58]. These social medical insurance schemes can cover at least 90% of Chinese, enabling more Chinese COPD patients to afford treatment. In 2006, the Chinese government published the “State Council Guidance on Development Urban Community Health Services”, which greatly promoted community-based primary care in China and reduced the risk of death from COPD [59]. With the reformation of medical education in China, there are more and better healthcare professionals to help patients with COPD [60]. Moreover, the rapid urbanization process (the urban population increased from 26.4% in 1990 to over 50% in 2011 [61]) has helped health services become more accessible.

Air pollution, both indoor and outdoor, is an important risk factor for COPD in China [62,63]. Indoor air pollution from domestic biomass combustion is considered to be the most important risk factor for COPD in developing countries [64]. In order to reduce indoor air pollution in China, many measures have been taken to improve the stove [65]. With rapid urbanization and economic development, cleaner energy sources, such as natural gas and electricity, have become more affordable and accessible [66]. Previous research has shown that indoor air pollution in China has declined since 2000 [67,68]. With the implementation of measures such as the construction of a natural gas pipeline network and coverage of terminal power grid in rural areas, indoor air pollution in China can be expected to decline in the future [69]. Outdoor air pollution, such as SO2, NO2, PM10, PM2.5, etc., is also an important risk factor for COPD in China. The Chinese government has implemented a number of measures to reduce PM10 and SO2, including more rigorous PM emission standard for power plants [70] and the application of fabric filters and electrostatic precipitators in large factories [71]. Under the influence of the above measures, SO2 and PM10 have decreased in the past 20 years [72,73], which has effectively reduced the risk of COPD mortality in China. However, due to the industrialization process and the increase in motor vehicles as a result of economic development in China, PM2.5 and NO2 have shown a significant upward trend in the last two decades [73,74,75]. We need to focus more attention on the reduction of NO2 and PM2.5 to prevent COPD in the future. As for the U.S., air pollution has declined only slightly there in the past two decades [76,77]. However, due to the huge impact of the rapidly increased smoking prevalence in the U.S. before 1965, the period RRs in the U.S. increased from 1992 to 2017.

Cohort effects represent early life exposure to socioeconomic, behavioral, and environmental factors. Economic development, and the related environmental and cultural changes, may be the main reasons for the downward trends in cohort RRs in China and the U.S. Previous studies have shown that environmental factors in the uterus and early childhood have far-reaching impact on the development of lung function [78]. Therefore, better maternal and perinatal health care as well as improvements in early childhood environment will contribute to reduced risk of death from COPD in adulthood. In addition, a better childhood environment is associated with less exposure to infections in early life and reduced risk of COPD in adulthood [79]. The promotion of education has also been thought to reduce COPD mortality [80], notably in China [81]. In later generations, better nutrition and better awareness of COPD-related knowledge may therefore also play an important role in reducing COPD mortality.

The slowdown in the downward trends in cohort RRs in China and the U.S. can be attributed to different reasons. In the U.S., the downward trend in cohort RRs slowed down in cohorts 1953–1957 and 1958–1962. In the context of the Cold War, the U.S. suffered an economic crisis in 1953, which led to a rapid decline in per capita income. The Vietnam War began in 1955 and involved a large number of U.S. personnel and state finances. These factors increased the risk of death in the U.S., including the risk of death from COPD. In China, the downward trend in cohort RRs slowed down on two nodes: cohort 1948–1952 and 1963–1967. For the cohort 1948–1952, the deceleration was mainly caused by the Liberation War and natural disaster. Air pollution caused by industrial development also increased the risk of COPD for this generation. The cohort 1963–1967 was affected by the Cultural Revolution and major economic systems, such as “the People’s Commune Movement” and “the Great Leap Forward”. They grew up in an environment of underdeveloped economy and poor nutrition, which increased the risk of death, including the risk of death from COPD.

This study still has some limitations. First, the problem of integrity and accuracy of COPD mortality data may lead to bias to some extent. Although GBD 2017 has undergone many corrections and adjustment steps to enhance data comparability, including mapping across different revisions and national variants of ICD as well as redistribution of deaths assigned to causes through garbage code redistribution, it is still difficult to thoroughly avoid bias. Second, the APC framework uses the population as a unit of observation and analysis, which may cause ecological fallacy. Therefore, the relevant hypotheses proposed in this study need to be further confirmed in future individualized research.

## 5. Conclusions

This study investigated the long-term trends in COPD mortality in China and the U.S. from 1992 to 2017 using the IE method of the APC model. From 1992 to 2017, the RRs of COPD mortality increased continuously in the U.S., while they decreased continuously in China. The increased RRs of COPD mortality in the U.S. were mainly attributable to the increased smoking prevalence before 1965, while the decreased RRs of COPD mortality in China were mainly attributable to improvements in medical technology and more accessible health services as well as reduced air pollution. Reducing tobacco consumption may be the most effective and feasible way to prevent COPD in China. However, with the improving smoking behavior in China and the U.S., we also need to pay more attention to COPD in nonsmokers in the future.

## Appendix A

**Table A1:** APC model analysis results of COPD mortality in China, 1992–2017.

Variables	Male			Female		
	Coef (95%CI)	RR (95%CI)	*p*	Coef (95%CI)	RR (95%CI)	*p*
Intercept	4.47 (4.36, 4.58)		0.00	3.99 (3.84, 4.15)		0.00
Age						
40–44	−2.03 (−2.27, −1.79)	1.00	0.00	−2.01 (−2.30, −1.72)	1.00	0.00
45–49	−1.62 (−1.81, −1.44)	1.50 (1.58, 1.42)	0.00	−1.52 (−1.74, −1.30)	1.63 (1.75, 1.52)	0.00
50–54	−1.01 (−1.16, −0.87)	2.76 (3.04, 2.50)	0.00	−1.02 (−1.19, −0.85)	2.69 (3.03, 2.39)	0.00
55–59	−0.46 (−0.57, −0.36)	4.80 (5.48, 4.20)	0.00	−0.53 (−0.66, −0.40)	4.39 (5.15, 3.75)	0.00
60–64	0.34 (0.26, 0.41)	10.68 (12.60, 9.04)	0.00	0.27 (0.18, 0.37)	9.78 (11.99, 8.05)	0.00
65–69	0.92 (0.86, 0.98)	19.11 (22.86, 15.98)	0.00	0.89 (0.81, 0.97)	18.17 (22.48, 14.66)	0.00
70–74	1.70 (1.64, 1.76)	41.75 (49.88, 34.95)	0.00	1.68 (1.59, 1.76)	40.04 (49.11, 32.39)	0.00
75–79	2.17 (2.09, 2.25)	66.79 (78.54, 56.79)	0.00	2.24 (2.13, 2.35)	70.09 (84.28, 58.23)	0.00
Period						
1992	0.38 (0.30, 0.45)	1.00	0.00	0.57 (0.48, 0.67)	1.00	0.00
1997	0.23 (0.18, 0.28)	0.87 (0.89, 0.85)	0.00	0.37 (0.31, 0.44)	0.82 (0.84, 0.79)	0.00
2002	0.07 (0.03, 0.11)	0.74 (0.76, 0.71)	0.00	0.11 (0.07, 0.16)	0.63 (0.66, 0.60)	0.00
2007	−0.18 (−0.22, −0.13)	0.57 (0.59, 0.56)	0.00	−0.24 (−0.30, −0.18)	0.44 (0.46, 0.43)	0.00
2012	−0.26 (−0.32, −0.21)	0.53 (0.53, 0.52)	0.00	−0.39 (−0.47, −0.31)	0.38 (0.39, 0.37)	0.00
2017	−0.24 (−0.32, −0.16)	0.54 (0.54, 0.54)	0.00	−0.42 (−0.53, −0.32)	0.37 (0.37, 0.37)	0.00
Cohort						
1913–1917	0.65 (0.53, 0.77)	1.00	0.00	0.68 (0.52, 0.84)	1.00	0.00
1918–1922	0.65 (0.55, 0.75)	1.00 (1.02, 0.98)	0.00	0.66 (0.52, 0.79)	0.98 (1.00, 0.95)	0.00
1923–1927	0.64 (0.55, 0.74)	0.99 (1.02, 0.97)	0.00	0.60 (0.48, 0.73)	0.92 (0.95, 0.89)	0.00
1928–1932	0.54 (0.45, 0.64)	0.90 (0.92, 0.87)	0.00	0.53 (0.40, 0.65)	0.86 (0.88, 0.83)	0.00
1933–1937	0.43 (0.32, 0.53)	0.80 (0.82, 0.79)	0.00	0.43 (0.29, 0.57)	0.78 (0.79, 0.76)	0.00
1938–1942	0.25 (0.13, 0.36)	0.67 (0.67, 0.66)	0.00	0.29 (0.13, 0.44)	0.68 (0.68, 0.67)	0.00
1943–1947	0.08 (−0.06, 0.22)	0.57 (0.56, 0.57)	0.26	0.13 (−0.06, 0.31)	0.58 (0.56, 0.59)	0.18
1948–1952	0.04 (−0.13, 0.20)	0.54 (0.52, 0.57)	0.65	0.06 (−0.16, 0.28)	0.54 (0.51, 0.57)	0.59
1953–1957	−0.20 (−0.40, 0.01)	0.43 (0.39, 0.47)	0.06	−0.19 (−0.47, 0.08)	0.42 (0.37, 0.47)	0.17
1958–1962	−0.54 (−0.82, −0.26)	0.30 (0.26, 0.36)	0.00	−0.56 (−0.94, −0.18)	0.29 (0.23, 0.36)	0.00
1963–1967	−0.51 (−0.86, −0.16)	0.31 (0.25, 0.40)	0.00	−0.55 (−1.03, −0.08)	0.29 (0.21, 0.40)	0.02
1968–1972	−0.82 (−1.32, −0.31)	0.23 (0.16, 0.34)	0.00	−0.86 (−1.55, −0.17)	0.21 (0.13, 0.36)	0.02
1973–1977	−1.22 (−2.34, −0.10)	0.15 (0.06, 0.42)	0.03	−1.20 (−2.73, 0.32)	0.15 (0.04, 0.60)	0.12
AIC	7.67			7.04		
BIC	−78.85			−86.69		
Deviance	14.06			6.21		

**Table A2:** APC model analysis results of COPD mortality in the U.S., 1992–2017.

Variables	Male			Female		
	Coef (95%CI)	RR (95%CI)	*p*	Coef (95%CI)	RR (95%CI)	*p*
Intercept	3.66 (3.52,3.79)		0.00	3.39 (3.25,3.53)		0.00
Age						
40–44	−2.50 (−3.00, −2.00)	1.00	0.00	−2.47 (−3.04, −1.91)	1.00	0.00
45–49	−1.68 (−1.99, −1.36)	2.27 (2.75, 1.90)	0.00	−1.59 (−1.94, −1.24)	2.42 (1.95, 3.00)	0.00
50–54	−0.85 (−1.08, −0.62)	5.21 (6.82, 3.97)	0.00	−0.73 (−0.99, −0.48)	5.68 (4.18, 7.77)	0.00
55–59	−0.09 (−0.26, 0.09)	11.13 (15.49, 8.08)	0.32	−0.04 (−0.23, 0.16)	11.40 (7.92,16.61)	0.71
60–64	0.58 (0.44, 0.71)	21.76 (31.19, 15.03)	0.00	0.57 (0.42, 0.71)	20.82 (13.74, 31.82)	0.00
65–69	1.11 (1.00, 1.21)	36.97 (54.60, 24.78)	0.00	1.06 (0.94, 1.17)	34.08 (21.76, 53.52)	0.00
70–74	1.55 (1.45, 1.64)	57.40 (85.63, 38.09)	0.00	1.46 (1.36, 1.56)	50.73 (32.14, 81.45)	0.00
75–79	1.89 (1.79, 1.99)	80.64 (120.3, 54.05)	0.00	1.75 (1.65, 1.85)	68.07 (42.95, 108.85)	0.00
Period						
1992	−0.27 (−0.39, −0.15)	1.00	0.00	−0.46 (−0.59, −0.32)	1.00	0.00
1997	−0.15 (−0.23, −0.06)	1.13 (1.17, 1.09)	0.00	−0.22 (−0.32, −0.12)	1.26 (1.22, 1.31)	0.00
2002	−0.02 (−0.08, 0.05)	1.28 (1.36, 1.22)	0.64	−0.02 (−0.10, 0.06)	1.55 (1.46, 1.63)	0.65
2007	0.04 (−0.03, 0.11)	1.36 (1.43, 1.30)	0.26	0.09 (0.01, 0.17)	1.73 (1.63, 1.82)	0.02
2012	0.13 (0.04, 0.22)	1.49 (1.54, 1.45)	0.00	0.23 (0.13, 0.32)	1.98 (1.90, 2.05)	0.00
2017	0.26 (0.15, 0.37)	1.70 (1.72, 1.68)	0.00	0.37 (0.25, 0.49)	2.29 (2.25, 2.32)	0.00
Cohort						
1913–1917	0.80 (0.62, 0.98)	1.00	0.00	0.64 (0.43, 0.84)	1.00	0.00
1918–1922	0.66 (0.52, 0.81)	0.87 (0.90, 0.84)	0.00	0.61 (0.44, 0.77)	0.97 (0.93, 1.01)	0.00
1923–1927	0.55 (0.42, 0.67)	0.78 (0.82, 0.73)	0.00	0.54 (0.39, 0.68)	0.90 (0.85, 0.96)	0.00
1928–1932	0.40 (0.28, 0.53)	0.67 (0.71, 0.64)	0.00	0.43 (0.30, 0.57)	0.82 (0.76, 0.88)	0.00
1933–1937	0.24 (0.10, 0.37)	0.57 (0.59, 0.54)	0.00	0.30 (0.15, 0.45)	0.72 (0.68, 0.76)	0.00
1938–1942	0.08 (−0.06, 0.23)	0.49 (0.51, 0.47)	0.27	0.14 (−0.02, 0.30)	0.61 (0.58, 0.64)	0.09
1943–1947	−0.07 (−0.25, 0.11)	0.42 (0.42, 0.42)	0.48	−0.03 (−0.23, 0.16)	0.51 (0.51, 0.52)	0.75
1948–952	−0.21 (−0.43, 0.01)	0.36 (0.35, 0.38)	0.06	−0.25 (−0.49, −0.01)	0.41 (0.43, 0.40)	0.04
1953−1957	−0.26 (−0.52, 0.01)	0.35 (0.32, 0.38)	0.06	−0.34 (−0.62, −0.05)	0.38 (0.41, 0.35)	0.02
1958−1962	−0.32 (−0.64, 0.01)	0.33 (0.28, 0.38)	0.06	−0.32 (−0.66, 0.02)	0.38 (0.44, 0.34)	0.07
1963−1967	−0.46 (−0.88, −0.03)	0.28 (0.22, 0.36)	0.04	−0.39 (−0.83, 0.04)	0.36 (0.45, 0.28)	0.08
1968−1972	−0.67 (−1.31, −0.04)	0.23 (0.15, 0.36)	0.04	−0.58 (−1.22, 0.06)	0.30 (0.46, 0.19)	0.08
1973−1977	−0.76 (−2.14, 0.62)	0.21 (0.06, 0.70)	0.28	−0.74 (−2.21, 0.72)	0.25 (0.89, 0.07)	0.32
AIC	6.51			6.26		
BIC	−92.21			−92.00		
Deviance	0.70			0.91		

## References

[1] Kim Y.S. (2017). Definition and Epidemiology of COPD. COPD: Heterogeneity and Personalized Treatment.

[2] Lopez-Campos J.L., Tan W., Soriano J.B. (2016). Global burden of COPD. Respirology.

[3] Yang G., Wang Y., Zeng Y., Gao G.F., Liang X., Zhou M., Wan X., Yu S., Jiang Y., Naghavi M. (2013). Rapid health transition in China, 1990–2010: Findings from the Global Burden of Disease Study 2010. Lancet.

[4] Centers for Disease Control and Prevention (2012). Chronic obstructive pulmonary disease among adults—United States, 2011. MMWR. Morb. Mortal. Wkly. Rep..

[5] Rawla P., Sunkara T., Gaduputi V. (2019). Epidemiology of Pancreatic Cancer: Global Trends, Etiology and Risk Factors. World J. Oncol..

[6] Rawla P., Sunkara T., Muralidharan P., Raj J.P. (2018). Update in global trends and aetiology of hepatocellular carcinoma. Contemp. Oncol..

[7] Rawla P., Barsouk A. (2019). Epidemiology of gastric cancer: Global trends, risk factors and prevention. Prz. Gastroenterol..

[8] Ren Y., Zhang M., Luo X., Zhao J., Yin L., Pang C., Feng T., Wang S., Wang B., Zhang H. (2017). Secular trend of the leading causes of death in China from 2003 to 2013. Afr. Health Sci..

[9] Institute for Health Metrics and Evaluation GBD Results Tool 2017. http://ghdx.healthdata.org/gbd-results-tool.

[10] Ford E.S., Croft J.B., Mannino D.M., Wheaton A.G., Zhang X.Y., Giles W.H. (2013). COPD Surveillance-United States, 1999–2011. Chest.

[11] Wang C., Xu J., Yang L., Xu Y., Zhang X., Bai C., Kang J., Ran P., Shen H., Wen F. (2018). Prevalence and risk factors of chronic obstructive pulmonary disease in China (the China Pulmonary Health [CPH] study): A national cross-sectional study. Lancet.

[12] Reilly K.H., Gu D., Duan X., Wu X., Chen C.S., Huang J., Kelly T.N., Chen J., Liu X., Yu L. (2008). Risk factors for chronic obstructive pulmonary disease mortality in Chinese adults. Am. J. Epidemiol..

[13] Qiu H., Tan K., Long F.Y., Wang L.Y., Yu H.Y., Deng R., Long H., Zhang Y.L., Pan J.P. (2018). The Burden of COPD Morbidity Attributable to the Interaction between Ambient Air Pollution and Temperature in Chengdu, China. Int. J. Environ. Res. Public Health.

[14] Brown D.W., Croft J.B., Greenlund K.J., Giles W.H. (2010). Trends in Hospitalization with Chronic Obstructive Pulmonary Disease—United States, 1990—2005. COPD.

[15] Yin P., Wang H., Vos T., Li Y., Liu S., Liu Y., Liu J., Wang L., Naghavi M., Murray C.J. (2016). A Subnational Analysis of Mortality and Prevalence of COPD in China from 1990 to 2013: Findings from the Global Burden of Disease Study 2013. Chest.

[16] Ford E.S. (2015). Trends in Mortality from COPD among Adults in the United States. Chest.

[17] GBD 2017 Mortality Collaborators (2018). Global, regional, and national age-sex-specific mortality for 282 causes of death in 195 countries and territories, 1980–2017: A systematic analysis for the Global Burden of Disease Study 2017. Lancet.

[18] Zhou M., Wang H., Zhu J., Chen W., Wang L., Liu S., Li Y., Wang L., Liu Y., Yin P. (2016). Cause-specific mortality for 240 causes in China during 1990–2013: A systematic subnational analysis for the Global Burden of Disease Study 2013. Lancet.

[19] GBD 2015 Chronic Respiratory Disease Collaborators (2017). Global, regional, and national deaths, prevalence, disability-adjusted life years, and years lived with disability for chronic obstructive pulmonary disease and asthma, 1990–2015: A systematic analysis for the Global Burden of Disease Study 2015. Lancet Respir. Med..

[20] Yang Y. (2008). Trends in US adult chronic disease mortality, 1960–1999: Age, period, and cohort variations. Demography.

[21] Yang Y., Land K.C. (2016). Age-Period-Cohort Analysis: New Models, Methods, and Empirical Applications.

[22] Mason K.O., Mason W.M., Winsborough H.H., Poole W.K. (1973). Some methodological issues in cohort analysis of archival data. Am. Sociol. Rev..

[23] Yang Y., Schulhofer-Wohl S., Fu W.J.J., Land K.C. (2008). The intrinsic estimator for age-period-cohort analysis: What it is and how to use it. Am. J. Sociol..

[24] Yang Y., Fu W.J.J., Land K.C. (2004). A methodological comparison of age-period-cohort models: The intrinsic estimator and conventional generalized linear models. Sociol. Methodol..

[25] Luo L. (2013). Assessing validity and application scope of the intrinsic estimator approach to the age-period-cohort problem. Demography.

[26] Tantucci C., Modina D. (2012). Lung function decline in COPD. Int. J. Chronic Obstr. Pulm. Dis..

[27] Makris D., Moschandreas J., Damianaki A., Ntaoukakis E., Siafakas N.M., Emili J.M., Tzanakis N. (2007). Exacerbations and lung function decline in COPD: New insights in current and ex-smokers. Respir. Med..

[28] Stanojevic S., Wade A., Stocks J., Hankinson J., Coates A.L., Pan H., Rosenthal M., Corey M., Lebecque P., Cole T.J. (2008). Reference ranges for spirometry across all ages—A new approach. Am. J. Respir. Crit. Care.

[29] Sharma G., Hanania N.A., Shim Y., Shim M. (2009). The aging immune system and its relationship to the development of chronic obstructive pulmonary disease. Proc. Am. Thorac. Soc..

[30] Lin H.H., Murray M., Cohen T., Colijn C., Ezzati M. (2008). Effects of smoking and solid-fuel use on COPD, lung cancer, and tuberculosis in China: A time-based, multiple risk factor, modelling study. Lancet.

[31] Lam K.B.H., Yin P., Jiang C.Q., Zhang W.S., Adab P., Miller M.R., Thomas G.N., Ayres J.G., Lam T.H., Cheng K.K. (2012). Past dust and GAS/FUME exposure and COPD in Chinese: The Guangzhou Biobank Cohort Study. Respir. Med..

[32] United Nations Department of Economic and Social Affairs Interactive Data—Profiles of Ageing 2017. https://population.un.org/ProfilesOfAgeing2017/index.html.

[33] Anderson R.N., Miniño A.M., Hoyert D.L., Rosenberg H.M. (2001). Comparability of cause of death between ICD-9 and ICD-10: Preliminary estimates. Natl. Vital Stat. Rep..

[34] Mannino D.M., Homa D.M., Akinbami L.J., Ford E.S., Redd S.C.J. (2002). Chronic obstructive pulmonary disease surveillance-United States, 1971–2000. Respir. Care.

[35] Centers for Disease Control and Prevention (1999). Tobacco use—United States, 1900–1999. MMWR. Morb. Mortal. Wkly. Rep..

[36] Giovino G.A., Schooley M.W., Zhu B.P., Chrismon J.H., Tomar S.L., Peddicord J.P., Merritt R.K., Husten C.G., Eriksen M.P. (1994). Surveillance for selected tobacco-use behaviors—United States, 1900–1994. MMWR CDC Surveill Summ.

[37] Kazerouni N., Alverson C.J., Redd S.C., Mott J.A., Mannino D.M. (2004). Sex differences in COPD and lung cancer mortality trends—United States, 1968–1999. J. Women’s Health.

[38] Ma J., Ward E.M., Siegel R.L., Jemal A. (2015). Temporal Trends in Mortality in the United States, 1969–2013. JAMA.

[39] Wellmann K.F. (1964). Smoking and Health. On the Report of the Advisory Committee to the Surgeon General of the Public Health Service. Dtsch. Med. Wochenschr..

[40] Wang T.W., Asman K., Gentzke A.S., Cullen K.A., Holder-Hayes E., Reyes-Guzman C., Jamal A., Neff L., King B.A. (2018). Tobacco Product Use Among Adults—United States, 2017. MMWR. Morb. Mortal. Wkly. Rep..

[41] Weng X.Z., Hong Z.G., Chen D.Y. (1987). Smoking prevalence in Chinese aged 15 and above. Chin. Med. J..

[42] Yang G., Fan L., Tan J., Qi G., Zhang Y., Samet J.M., Taylor C.E., Becker K., Xu J. (1999). Smoking in China: Findings of the 1996 National Prevalence Survey. JAMA.

[43] Wang M., Luo X., Xu S., Liu W., Ding F., Zhang X., Wang L., Liu J., Hu J., Wang W. (2019). Trends in smoking prevalence and implication for chronic diseases in China: Serial national cross-sectional surveys from 2003 to 2013. Lancet Respir. Med..

[44] Qian J., Cai M., Gao J., Tang S., Xu L., Critchley J.A. (2010). Trends in smoking and quitting in China from 1993 to 2003: National Health Service Survey data. Bull. World Health Organ..

[45] The World Bank Database, Smoking Prevalence, Total (Ages 15+). https://data.worldbank.org/indicator/SH.PRV.SMOK?year_low_desc=true.

[46] Sun Y.L., Sin D.D. (2018). Crisis of COPD in China: The future is now. Lancet Respir. Med..

[47] Zhou Y., Wang C., Yao W., Chen P., Kang J., Huang S., Chen B., Ni D., Wang X., Wang D.J. (2009). COPD in Chinese nonsmokers. Eur. Respir. J..

[48] Syamlal G., Mazurek J.M. (2019). Chronic Obstructive Pulmonary Disease Prevalence Among Adults Who Have Never Smoked, by Industry and Occupation—United States, 2013–2017. MMWR. Morb. Mortal. Wkly. Rep..

[49] Mannino D.M., Gagnon R.C., Petty T.L., Lydick E.J. (2000). Obstructive lung disease and low lung function in adults in the United States: Data from the National Health and Nutrition Examination Survey, 1988–1994. Arch. Intern. Med..

[50] Celli B.R., Halbert R.J., Isonaka S., Schau B. (2003). Population impact of different definitions of airway obstruction. Eur. Respir. J..

[51] Gómez F.P., Rodriguez-Roisin R.J. (2002). Global Initiative for Chronic Obstructive Lung Disease (GOLD) guidelines for chronic obstructive pulmonary disease. Curr. Opin. Pulm. Med..

[52] Martinez C.H., Mannino D.M., Jaimes F.A., Curtis J.L., Han M.K., Hansel N.N., Diaz A.A. (2015). Undiagnosed Obstructive Lung Disease in the United States Associated Factors and Long-term Mortality. Ann. Am. Thorac. Soc..

[53] Halbert R.J., Natoli J.L., Gano A., Badamgarav E., Buist A.S., Mannino D.M. (2006). Global burden of COPD: Systematic review and meta-analysis. Eur. Respir. J..

[54] Zielinski J., Bednarek M., Know the Age of Your Lung Study Group (2001). Early detection of COPD in a high-risk population using spirometric screening. Chest.

[55] Zhong N., Wang C., Yao W., Chen P., Kang J., Huang S., Chen B., Wang C., Ni D., Zhou Y. (2007). Prevalence of chronic obstructive pulmonary disease in China—A large, population-based survey. Am. J. Respir. Crit. Care.

[56] Zheng J., Yang J., Zhou X., Zhao L., Hui F., Wang H., Bai C., Chen P., Li H., Kang J. (2014). Roflumilast for the treatment of COPD in an Asian population: A randomized, double-blind, parallel-group study. Chest.

[57] Zheng J.P., Yang L., Wu Y.M., Chen P., Wen Z.G., Huang W.J., Shi Y., Wang C.Z., Huang S.G., Sun T.Y. (2007). The efficacy and safety of combination salmeterol (50 μg)/fluticasone propionate (500 μg) inhalation twice daily via Accuhaler in Chinese patients with COPD. Chest.

[58] Wang H.J. (2009). A dilemma of Chinese healthcare reform: How to re-define government roles?. China Econ. Rev..

[59] Wang H., Gusmano M.K., Cao Q.J. (2011). An evaluation of the policy on community health organizations in China: Will the priority of new healthcare reform in China be a success?. Health Policy.

[60] Xu D., Sun B., Wan X., Ke Y. (2010). Reformation of medical education in China. Lancet.

[61] Zhang C.G., Lin Y. (2012). Panel estimation for urbanization, energy consumption and CO_2_ emissions: A regional analysis in China. Energy Policy.

[62] Guan W.-J., Zheng X.-Y., Chung K.F., Zhong N. (2016). Impact of air pollution on the burden of chronic respiratory diseases in China: time for urgent action. Lancet.

[63] Song Q.K., Christiani D.C., Wang X.R., Ren J. (2014). The Global Contribution of Outdoor Air Pollution to the Incidence, Prevalence, Mortality and Hospital Admission for Chronic Obstructive Pulmonary Disease: A Systematic Review and Meta-Analysis. Int. J. Environ. Res. Public Health.

[64] Salvi S., Barnes P.J. (2010). Is Exposure to Biomass Smoke the Biggest Risk Factor for COPD Globally?. Chest.

[65] Shuhua G., Kun H., Daixong Q., Smith K.R. (1993). One hundred million improved cookstoves in China: How was it done. World Dev..

[66] Mestl H.E., Aunan K., Seip H.M., Wang S., Zhao Y., Zhang D. (2007). Urban and rural exposure to indoor air pollution from domestic biomass and coal burning across China. Sci. Total Environ..

[67] Aunan K., Wang S.J. (2014). Internal migration and urbanization in China: Impacts on population exposure to household air pollution (2000–2010). Sci. Total Environ..

[68] Zhao B., Zheng H.T., Wang S.X., Smith K.R., Lu X., Aunan K., Gu Y., Wang Y., Ding D., Xing J. (2018). Change in household fuels dominates the decrease in PM2.5 exposure and premature mortality in China in 2005–2015. Proc. Natl. Acad. Sci. USA.

[69] Shen G.F. (2016). Changes from traditional solid fuels to clean household energies—Opportunities in emission reduction of primary PM2.5 from residential cookstoves in China. Biomass Bioenergy.

[70] Lei Y., Zhang Q., He K.B., Streets D.G. (2011). Primary anthropogenic aerosol emission trends for China, 1990–2005. Atmos. Chem. Phys..

[71] Wang S.X., Hao J.M. (2012). Air quality management in China: Issues, challenges, and options. J. Environ. Sci. China.

[72] Cheng Z., Jiang J.K., Fajardo O., Wang S.X., Hao J.M. (2013). Characteristics and health impacts of particulate matter pollution in China (2001–2011). Atmos. Environ..

[73] Chen B., Hong C., Kan H. (2004). Exposures and health outcomes from outdoor air pollutants in China. Toxicology.

[74] Liu M., Huang Y., Ma Z., Jin Z., Liu X., Wang H., Liu Y., Wang J., Jantunen M., Bi J. (2017). Spatial and temporal trends in the mortality burden of air pollution in China: 2004–2012. Environ. Int..

[75] Zhang Q., Geng G.N., Wang S.W., Richter A., He K.B. (2012). Satellite remote sensing of changes in NO_x_ emissions over China during 1996–2010. Chin. Sci. Bull..

[76] Hand J.L., Schichtel B.A., Malm W.C., Pitchford M.L. (2012). Particulate sulfate ion concentration and SO_2_ emission trends in the United States from the early 1990s through 2010. Atmos. Chem. Phys..

[77] Hart J.E., Yanosky J.D., Puett R.C., Ryan L., Dockery D.W., Smith T.J., Garshick E., Laden F. (2009). Spatial Modeling of PM_10_ and NO_2_ in the Continental United States, 1985–2000. Environ. Health Perspect..

[78] Postma D.S., Bush A., van den Berge M.J. (2015). Risk factors and early origins of chronic obstructive pulmonary disease. Lancet.

[79] Wedzicha J.A., Seemungal T.A. (2007). COPD exacerbations: Defining their cause and prevention. Lancet.

[80] Yin P., Zhang M., Li Y., Jiang Y., Zhao W.J. (2011). Prevalence of COPD and its association with socioeconomic status in China: Findings from China Chronic Disease Risk Factor Surveillance 2007. BMC Public Health.

[81] Tsang M.C.J. (2000). Education and national development in China since 1949: Oscillating policies and enduring dilemmas. China Rev..

